# Musashi-1 is the candidate of the regulator of hair cell progenitors during inner ear regeneration

**DOI:** 10.1186/s12868-017-0382-z

**Published:** 2017-08-16

**Authors:** Takahiro Wakasaki, Hiroaki Niiro, Siamak Jabbarzadeh-Tabrizi, Mitsuru Ohashi, Takashi Kimitsuki, Takashi Nakagawa, Shizuo Komune, Koichi Akashi

**Affiliations:** 1 Department of Otorhinolaryngology, Graduate School of Medical Sciences, Kyushu University, Fukuoka, Japan; 2 Department of Medicine and Biosystemic Science, Graduate School of Medical Sciences, Kyushu University, Fukuoka, Japan; 3 Department of Head and Neck Surgery, National Hospital Organization, Kyushu Cancer Center, 3-1-1 Notame, Miniami-ku, Fukuoka, 811-1395 Japan

**Keywords:** Musashi-1, Chick inner ear, Regeneration, cDNA microarray, Laser capture microdissection

## Abstract

**Background:**

Hair cell loss in the cochlea is caused by ototoxic drugs, aging, and environmental stresses and could potentially lead to devastating pathophysiological effects. In adult mammals, hair cell loss is irreversible and may result in hearing and balance deficits. In contrast, nonmammalian vertebrates, including birds, can regenerate hair cells through differentiation of supporting cells and restore inner ear function, suggesting that hair cell progenitors are present in the population of supporting cells.

**Results:**

In the present study, we aimed to identify novel genes related to regeneration in the chicken utricle by gene expression profiling of supporting cell and hair cell populations obtained by laser capture microdissection. The volcano plot identified 408 differentially expressed genes (twofold change, *p* = 0.05, Benjamini–Hochberg multiple testing correction), 175 of which were well annotated. Among these genes, we focused on Musashi-1 (MSI1), a marker of neural stem cells involved in Notch signaling, and the downstream genes in the Notch pathway. Higher expression of these genes in supporting cells compared with that in hair cells was confirmed by quantitative reverse transcription polymerase chain reaction. Immunohistochemistry analysis demonstrated that MSI1 was mainly localized at the basal side of the supporting cell layer in normal chick utricles. During the regeneration period following aminoglycoside antibiotic-induced damage of chicken utricles, the expression levels of MSI1, hairy and enhancer of split-5, and cyclin D1 were increased, and BrdU labeling indicated that cell proliferation was enhanced.

**Conclusions:**

The findings of this study suggested that MSI1 played an important role in the proliferation of supporting cells in the inner ear during normal and damaged conditions and could be a potential therapeutic target in the treatment of vestibular defects.

**Electronic supplementary material:**

The online version of this article (doi:10.1186/s12868-017-0382-z) contains supplementary material, which is available to authorized users.

## Background

In mammals, permanent inner ear disorders are commonly caused by the loss of sensory hair cells (HCs) due to aging, environmental stresses (e.g., acoustic trauma), and exposure to ototoxic drugs (e.g., cisplatin and aminoglycoside antibiotics) [1]. Following HC destruction, only a limited number of supporting cells (SCs) in the sensory epithelia can divide and differentiate into HCs; consequently, the recovery of inner ear function in adult mammals can only be partial [2]. In contrast, in nonmammalian vertebrates, damaged and lost HCs can be replaced by HCs differentiated from SCs, and vestibular functional activity can be completely restored after injury [3]. Thus, in the utricles of birds, HC regeneration from SCs occurs in both resting and damage-induced states [4], and differentially expressed genes in SCs may therefore be attractive therapeutic targets for the treatment of inner ear disorders. For example, previous studies have shown that Atoh1, a basic helix-loop-helix transcription factor involved in neuronal cell development, promotes the differentiation of SCs to HCs in the inner ear [5]. However, Atoh1 expression could result in the depletion of HC progenitor cells in the inner ear during regeneration [5], undermining the role of Atoh1 in HC regeneration. Therefore, other proteins should be investigated as candidates for the restoration of HC populations.

Recent progress in microarray and other high-throughput technologies has made it possible to measure the relative abundance of mRNA in several thousand genes simultaneously. However, one of the major problems in comprehensive gene expression profiling is preparation of high-purity samples not subjected to chemical or mechanical processing. In most previous studies, mRNA has been prepared from whole acoustic organs or the sensory epithelium, which contains both HCs and SCs, and isolation has been achieved by thermolysin incubation and mechanical separation [6]. However, these methods have several disadvantages; indeed, whole organs consist of several cell types (e.g., epithelial HCs and SCs, fibroblasts, endothelial cells, and blood cells from the underlying stroma), and chemical and mechanical stresses applied to the sensory epithelium can trigger cell repair and/or death signaling [7, 8]. As a result, these methods are not suitable for elucidation of gene expression in normal tissues.

To overcome these problems with sample preparation, laser capture microdissection (LCM) is now widely used to obtain target cell populations from specific microscopic regions with minimal damage [7]. For example, Ricardo et al. utilized LCM technology to isolate HCs and SCs from rat vestibules and extract mRNA from pooled samples for microarray analysis [8].

Their study demonstrated that LCM is a reliable technique for the isolation of cell subpopulations from the sensory epithelium and presented a standardized protocol of gene expression profiling in the normal inner ear. However, pooled samples may exhibit interindividual variation, and a recent study showed that a one animal-one chip approach provides stronger statistical significance for the data [9].

In the present study, we aimed to identify novel genes associated with vestibular regeneration by detection of highly expressed genes in chicken SCs, including HC progenitors, using a combination of LCM and microarray techniques. Among the 175 selected annotated genes, we focused on Musashi-1 (MSI1), a transcription factor that functions as a key signaling molecule in the Notch pathway and plays a critical role in the maintenance and differentiation of stem cells [10].

## Methods

### Animals and treatments

This study was conducted in accordance with Japanese guidelines for the use of experimental animals and was approved by the Animal Care and Use Committee of Kyushu University. White leghorns (Kyudo Co., Ltd., Kumamoto, Japan) 10–12 days post-hatching were used for gene expression profiling with microarray and reverse transcription polymerase chain reaction (RT-PCR). A model of ototoxic damage was created using chickens at 7 days post-hatching after intramuscular injection of gentamicin sulfate (300 mg/kg; Wako Pure Chemical Industries, Osaka, Japan) for 4 consecutive days in the morning. Control animals were injected with normal saline. Birds were sacrificed 2, 7, or 14 days after the last injection and analyzed for the expression of selected genes in the utricles. Some of these animals also received an intramuscular injection of BrdU (100 mg/kg; Merck KGaA, Darmstadt, Germany) 24 h before sacrifice and analyzed for BrdU incorporation by immunohistochemistry.

### Capture of HCs and SCs by LCM and extraction of total RNA

Chickens were anesthetized with diethyl ether and decapitated; then, temporal bones were removed, and individual utricles were dissected under a microscope (SMZ745; Nikon, Tokyo, Japan). The utricles were quickly embedded in Tissue-Tek OCT (Sakura Finetek Japan, Tokyo, Japan), frozen in liquid nitrogen-chilled isopentane for 15 s, and stored at −80 °C until use. Tissues were sectioned at 10-μm thickness with a Microm HM500 OM Cryostat (MICROM International GmbH, Walldorf, Germany) and melted onto nuclease-free PALM Membrane slides (PALM GmbH, Bernried, Germany). The slides were incubated sequentially in RNase-free 100% ethanol for 60 s, water for 120 s, 0.05% toluidine blue for 10 s, and water for 20 s. Slides were then air-dried for 60 s and transferred to the PALM MB III microdissection system (Carl Zeiss, Oberkochen, Germany). An LCM was performed by capturing the cytoplasm and nuclei of HCs and SCs from individual utricle samples into different caps according to the cell type using an Arcturus Pixcell II LCM Microdissection System (Arcturus Engineering, Mountain View, CA, USA). Total RNA was isolated from HC- and SC-enriched samples using a Picopure RNA Isolation Kit (Bucher Biotec AG, Basel, Switzerland) and DNase I (Qiagen, Hilden, Germany) according to the manufacturer’s instructions and was then used for microarray analysis and quantitative RT-PCR.

### cDNA microarray

Total RNA from each sample was subjected to two rounds of amplification using a MessageAmpII aRNA Amplification Kit and biotin-11-UTP (both from Applied Biosystems, Austin, TX, USA) according to the manufacturer’s instructions to obtain biotin-labeled antisense (a)RNA. Because the yield of total RNA was too low to measure absorbance, we could not check RNA quality of LCM samples. However, the quality and quantity of the amplified biotin-labeled aRNA (65–167 μg per sample; average, 124 μg), as determined using an Agilent 2100 Bioanalyzer (Agilent Technologies Inc., Santa Clara, CA, USA), were excellent. Fragmented labeled aRNA (15 μg) was hybridized to a GeneChip Chicken Genome Array (Affymetrix, Santa Clara, CA, USA) containing 32,773 transcripts corresponding to over 28,000 chicken genes. Array hybridization and scanning were performed using standard procedures according to the manufacturer’s instructions. Gene expression was calculated as the average intensity difference between perfectly matched and mismatched probe pairs using MAS 5.0 (Affymetrix). The list of expressed probes was imported into GeneSpring GX 7.3.1 (Agilent Technologies, Palo Alto, CA, USA) for further data characterization. Briefly, the imported raw data were normalized based on the near default order of data transformation (set measurements less than 0.01 to 0.01) per chip (the 50th percentile) and per gene (median). Then, genes were extracted using quality control procedures, which flagged the data present or marginal in at least 5 of 10 samples, detected raw values higher than 30 in at least 5 of 10 samples, and identified samples in each group with a standard deviation of less than 0.5. These data were hierarchically clustered using centered Pearson’s correlation and centroid linkage. A volcano plot of quality-controlled genes identified the genes with an absolute fold change of greater than 2 and a parametric p value of less than 0.05 (Benjamini–Hochberg false discovery rate multiple testing correction). The data were deposited in the NCBI Gene Expression Omnibus (GEO, http://www.ncbi.nlm.nih.gov/geo/; accession number GSE81461).

### Quantitative RT-PCR

To validate the microarray data and determine the expression level of selected genes in SCs after damage, we performed quantitative RT-PCR. cDNA was synthesized using a reverse transcription kit (Qiagen, Hilden, Germany) according to the manufacturer’s instructions and analyzed by polymerase chain reaction (PCR) using Power SYBR Green PCR master mix or TaqMan probe master mix and an ABI Prism 7500 sequence detection system (Applied Biosystems). The reaction was performed using the following conditions: 45 cycles of denaturation at 95 °C for 15 s and annealing at 60 °C for 60 min; the specificity of SYBR Green PCR was tested by dissociation curve analysis. Chicken *GAPDH* (SYBR Green) or rRNA 18S (TaqMan probe) genes were separately amplified in the same plate and used as an internal control. The primers for the amplification of selected genes (Additional file 1: Table S1) were designed using Primer 3 software and synthesized by GENENET (Fukuoka, Japan). The data were analyzed using Sequence Detector software (Applied Biosystems).

### Immunohistochemistry and imaging

All procedures were performed at room temperature; unless stated otherwise, probes were washed with phosphate-buffered saline (PBS) between steps and protected from direct light.

For staining of sections, temporal bones including utricles were dissected from 12-day-old chickens, fixed in 3.7% paraformaldehyde overnight, and incubated in 10% ethylenediaminetetraacetic acid (EDTA) in PBS for 3 days. Samples were then placed in Tissue-Tek OCT cryo-embedding medium, frozen in liquid nitrogen-chilled isopentane for 15 s, and stored at −80 °C until use. Tissues were sectioned at 16-μm thickness, as described above, and utricles were permeabilized in 1% Triton X-100 (Sigma-Aldrich, St. Louis, MO, USA) in PBS for 10 min. Tissue sections were blocked with 10% normal goat serum for 30 min, incubated with a mouse anti-MSI1 primary antibody (1:100; R&D Systems, Minneapolis, MN, USA) at 4 °C overnight, and then incubated with an Alexa Fluor 488-conjugated goat anti-mouse secondary antibody (1:1000; Invitrogen, Carlsbad, CA, USA) for 1 h. Sections were counterstained with rhodamine-phalloidin (Cytoskeleton, Denver, CO, USA) for 15 min to visualize F-actin in stereocilia bundles and DAPI (1 μg/mL; Sigma-Aldrich) for 10 min to visualize the nuclei, washed for 10 min, and mounted in Vectashield (Vector Laboratories, Burlingame, CA, USA).

Next, DNA synthesis in SCs was assessed during regeneration by whole mount staining. Damaged and control utricle explants were labeled with BrdU. Utricle explants were incubated at 37 °C with 500 µg/mL thermolysin (Sigma-Aldrich) for 30 min to remove other tissues from the sensory epithelium containing HCs and SCs. Utricles were then fixed in 3.7% formaldehyde for 30 min, permeabilized, and incubated with 1% Triton-X in 2 N HCl for 30 min at 37 °C to denature DNA. Samples were blocked with 10% normal goat serum in PBS for 60 min, incubated with a mouse anti-BrdU antibody (1:100; Merck KGaA), and then incubated with an Alexa Fluor 568-conjugated goat anti-mouse secondary antibody (1:100; Invitrogen). Samples were finally mounted in Vectashield, and images were obtained under a Nikon A1 confocal laser-scanning microscope (Nikon, Tokyo, Japan) and processed using Adobe Photoshop CS4 (Adobe Systems, San Jose, CA, USA). For BrdU-treated chickens, a series of confocal z-stack images of whole-mount utricle samples were obtained at 10× magnification and assembled into a single image by Adobe Photoshop, which was used to count BrdU-labeled cells in the sensory epithelium of the whole utricle. The experiments were performed at least four times.

### Protein extraction and immunoblotting

Damaged and undamaged utricles were treated with thermolysin at 37 °C for 30 min, and two utricles from the same chicken were homogenized and lysed with RIPA buffer containing protein inhibitors. Protein extraction and immunoblotting were performed as described previously using antibodies against MSI1 (1:500), cyclin D1 (1:1000; Lifespan, Seattle, WA, USA), and β-actin (1:2000; Sigma-Aldrich) followed by secondary anti-rabbit, anti-mouse, or anti-goat horseradish peroxidase (HRP)-conjugated IgG (1:15,000; Jackson ImmunoResearch Laboratories, West Grove, PA, USA). Protein bands were developed with an ECL Plus kit (GE Healthcare Bio-Sciences Corp, Piscataway, NJ, USA) and visualized using a Laser 3000 instrument (Fuji Film, Tokyo, Japan). Chemiluminescence intensity was quantified using ImageJ software (rsb.info.nih.gov/ij).

### Statistical analysis

Statistical analyses were performed using JMP (version 10) statistical software. The data are presented as the mean ± standard error of the mean based on at least four independent experiments. The differences between samples were evaluated by the Mann–Whitney *U*-test, and differences with *p* values of less than 0.05 were considered statistically significant.

## Results

### Microarray characterization

**Fig. 1:**
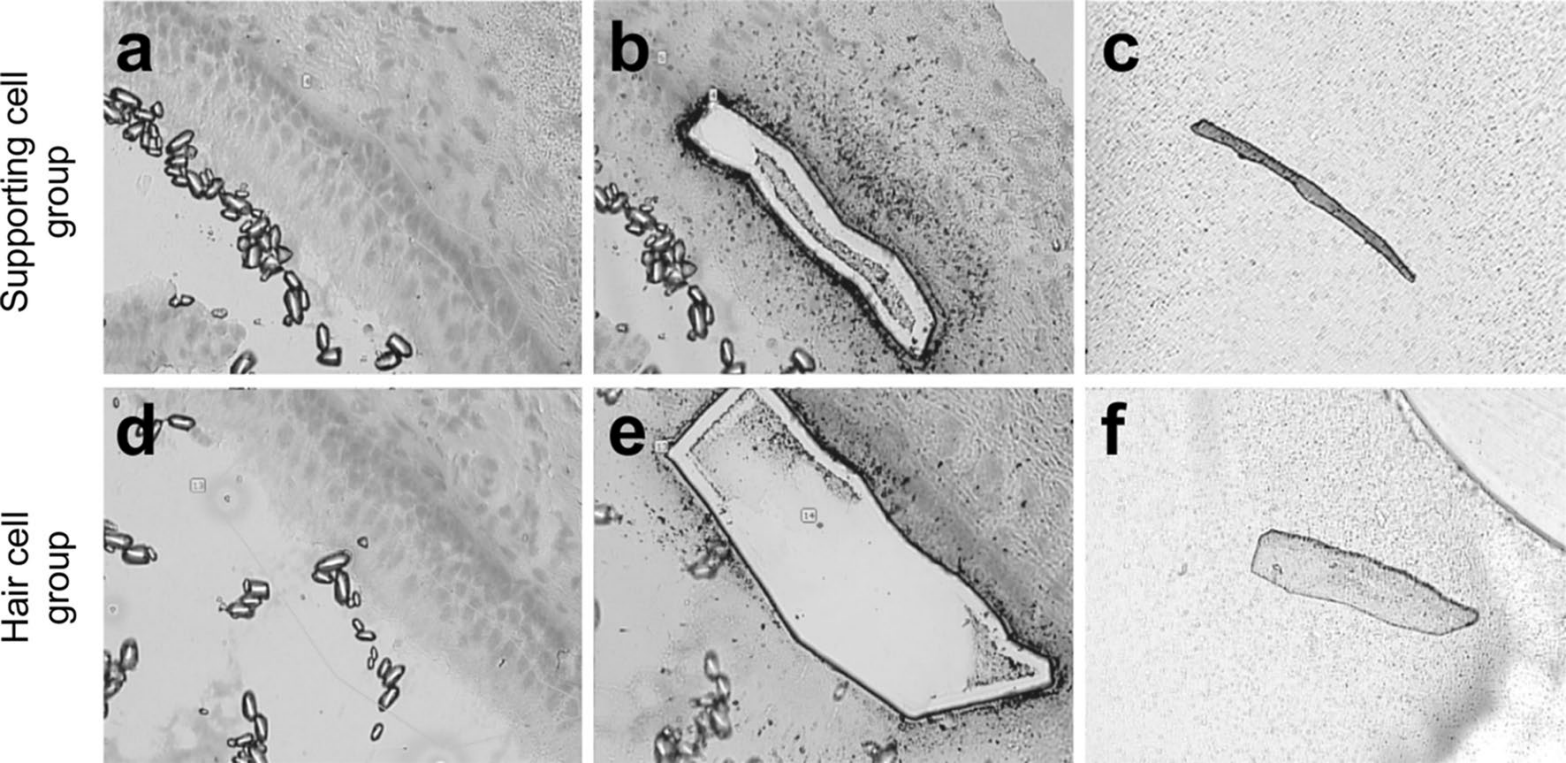
Large aperture laser capture microdissection of vestibular hair cells and supporting cells from chicken utricles. **a** Histological section of the utricle before microdissection. **b** The utricle with overlying microcapture film in situ after SC microdissection. **c** Captured SCs adherent to the cap. **d** The utricle after the removal of microcaptured HCs. **e** Histological section with overlying microcapture film in situ after HC microdissection. **f** Captured HCs adherent to the cap

Laser capture microdissection allowed successful selective acquisition of enriched HC and SC populations from a single utricle (Fig. 1), each containing approximately 4000 cells.

**Fig. 2:**
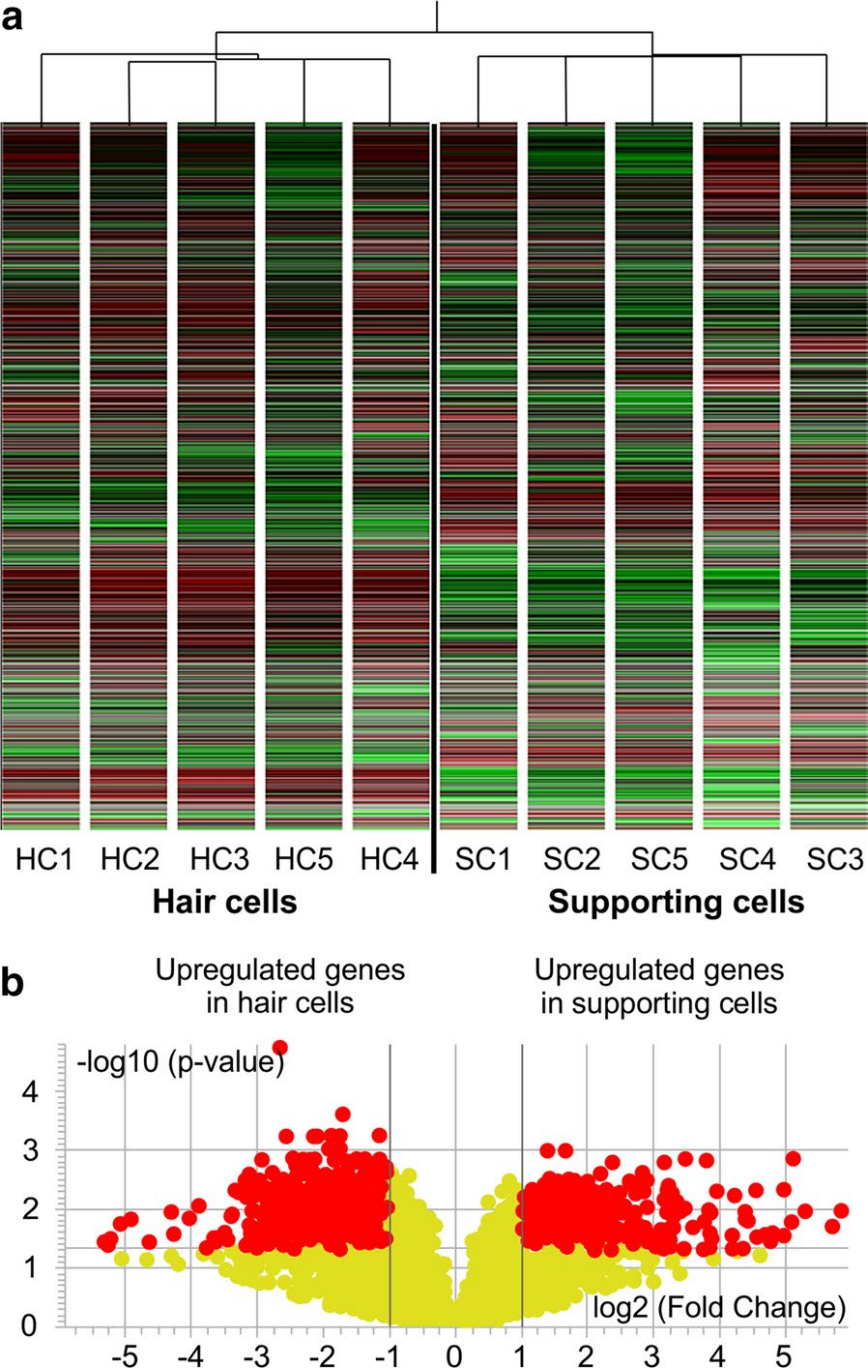
Identification of genes differentially expressed in hair cells and supporting cells. **a** Hierarchical clustering. **b** Volcano plot (fold change >2, *p* < 0.05; Benjamini–Hochberg multiple testing correction)

Amplified aRNA from each sample was subjected to microarray hybridization, and 12,794 extracted genes were analyzed by hierarchical clustering, which revealed distinct expression patterns in HC and SC samples (Fig. 2a).

### Differential expression analysis

**Fig. 3:**
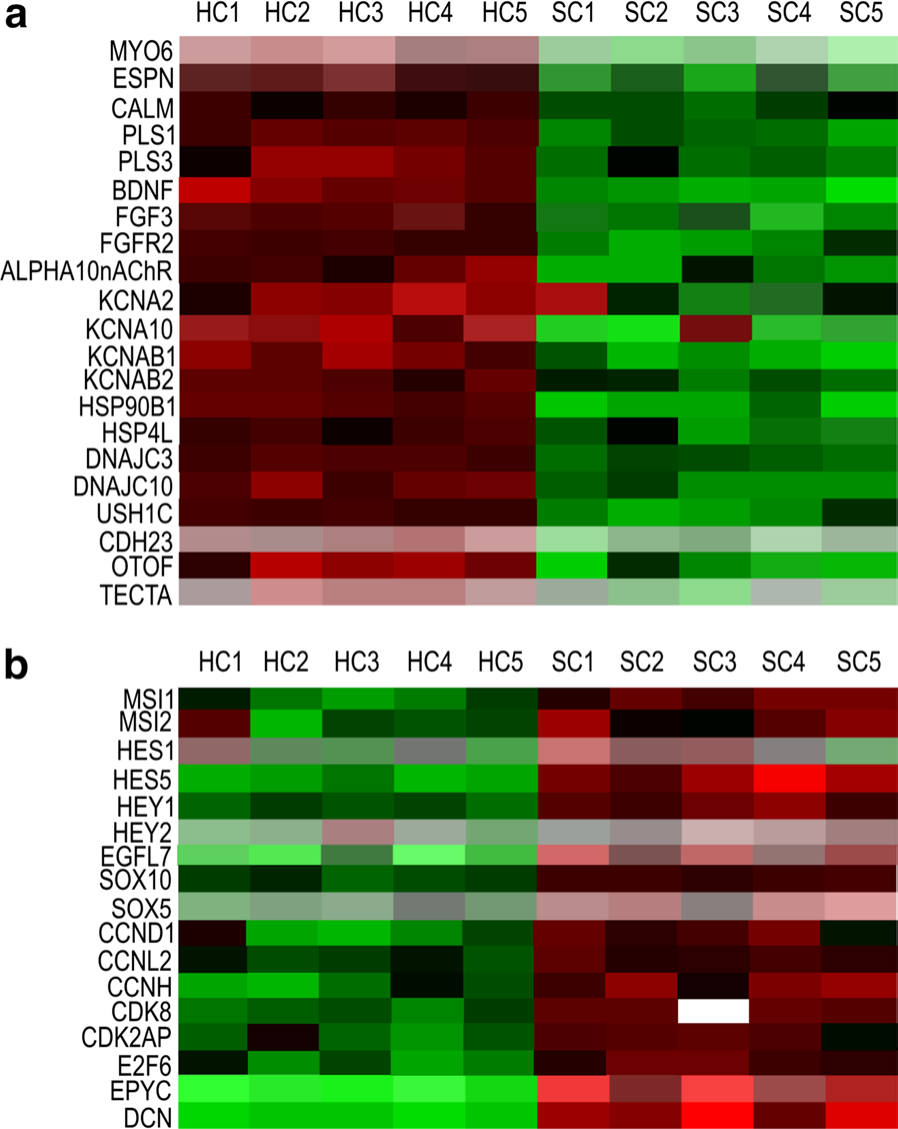
Heat maps of genes upregulated in hair cells (**a**) and supporting cells (**b**). *HC* hair cell; *SC* supporting cell; *numbers* indicate individual cell samples

A volcano plot of the identified quality-controlled genes (*p* < 0.05, fold change >2) is presented in Fig. 2b. In total, there were 1303 genes with more than twofold upregulated expression. There were 433 in the SC group and 870 in the HC group; among them, 148 and 376 genes, respectively, were annotated. A heat map was generated to show genes previously identified in HCs that were significantly upregulated in these cells (Fig. 3a).

Among these upregulated genes, we found genes encoding cytoskeletal proteins (ESPN and MYO6), neurotrophic and growth factors (BDNF and FGF3, respectively), a growth factor receptor (FGFR2), the alpha 10 subunit of nicotinic acetylcholine receptor (CHRNA10), several potassium voltage-gated channels (KCNA2, KCNA10, KCNAB1, and KCNAB2), a potassium large conductance calcium-activated channel (KCNMA1), a voltage-dependent calcium channel (CACNA1D), heat shock proteins (HSP90B1 and HSPA4L), and deafness-associated factors (USH1C, OTOF, and TECTA). Other significantly upregulated (≥fivefold) genes in the HC group are shown in Additional file 1: Table S2. Several well-known HC markers, including myosin VIIA (MYO7A) and protein atonal homolog 1 (ATOH1) were removed during quality control because of poor quality (insufficient flags) and expression levels or high standard deviations.

**Fig. 4:**
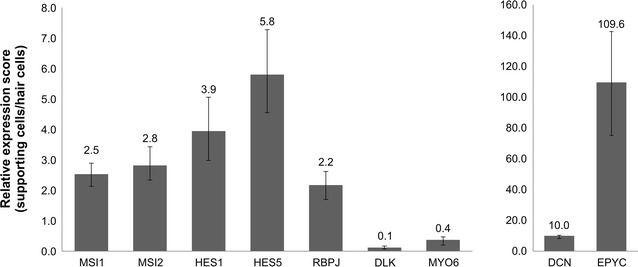
Quantitative RT-PCR analysis of the target genes to validate the microarray data

Genes with the highest upregulation in SCs compared with that in HCs encoded proteoglycans (EPYC and DCN; Fig. 3b). The expression of cell cycle-associated genes (CCND1, CCNL2, CCNH, CDK8, CDK2AP, and E2F6) was also higher in the SC group. The following genes encoding stem cell markers and related factors were also upregulated in SCs: Musashi (MSI1 and MSI2), genes downstream of Musashi in the Notch pathway (i.e., HES1, HES5, HEY1, HEY2, and EGFL7), and SRY-related HMG-box (SOX) family members (SOX10 and SOX5). Additional file 1: Table S3 shows all genes with higher expression in SCs (≥twofold) than in HCs. To confirm the microarray data, selected genes were validated by quantitative RT-PCR analysis of other HC and SC samples (Fig. 4).

Consistent with the microarray results, the HC marker MYO6 showed higher expression in HCs, while EPYC, DCN, CCND1, and the effectors of Notch signaling MSI1, MSI2, HES1, HES5, and RBPJ were upregulated in SCs.

### Upregulation of MSI1 expression in the sensory epithelium during SC proliferation after drug-induced damage

**Fig. 5:**
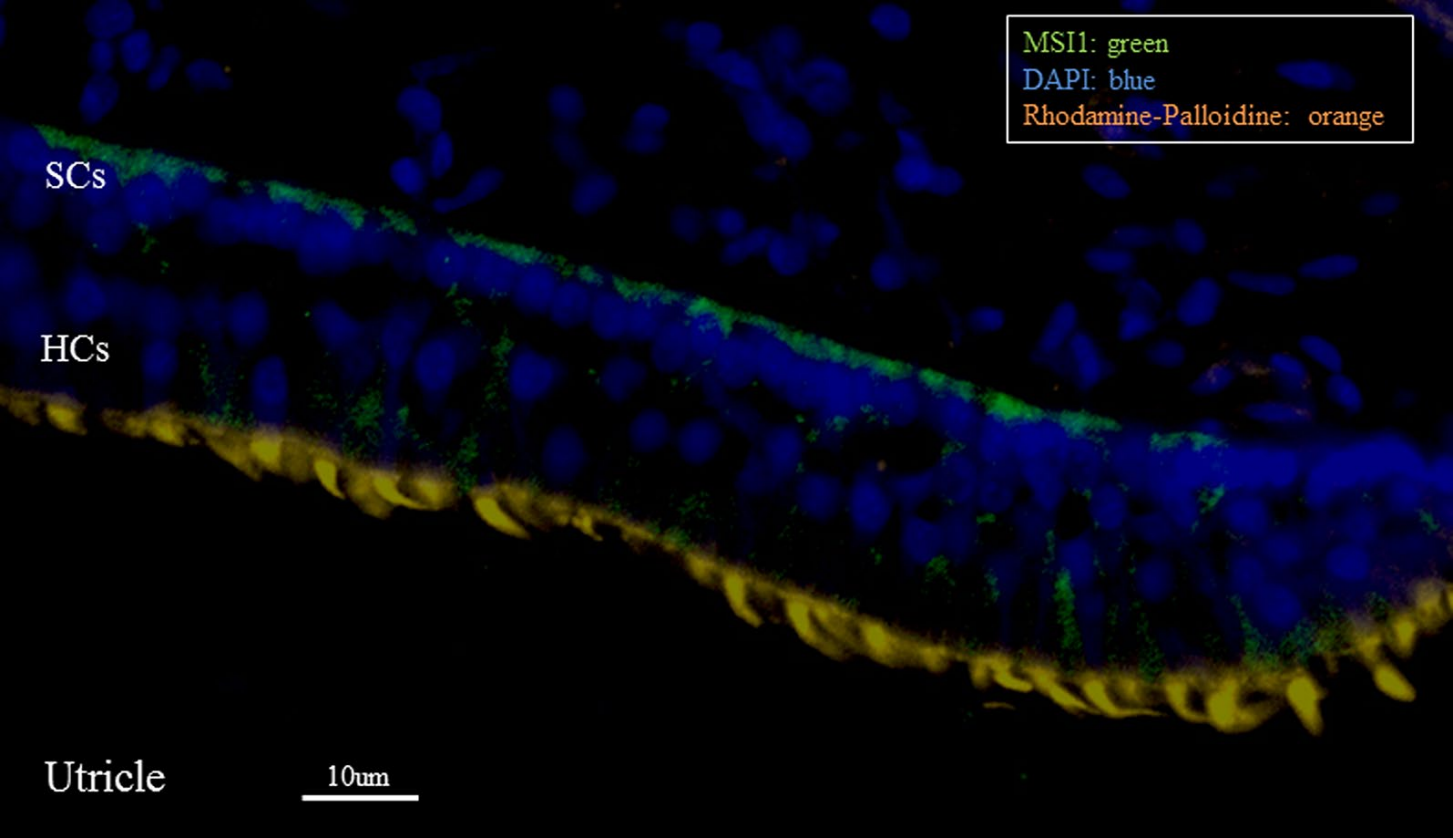
Expression of Musashi 1 in the utricle from normal 12-day-old chickens. Immunohistochemistry analysis showed that MSI1 was mainly located at the basal side of supporting cells in the utricle. *MSI* Musashi

Because MSI1 has been reported as a candidate maintenance gene for stem cells in several organs [11, 12], we hypothesized that MSI1 could have a role in the regenerative processes of the inner ear after ototoxic damage. To test this hypothesis, we treated chickens with aminoglycoside and compared DNA synthesis and the expression of MSI1 and related genes in the utricles of normal and damaged inner ears. Immunohistochemistry analysis demonstrated that MSI1 protein was mainly expressed at the basal side of the SC layer in utricles from normal 12-day-old chickens (Fig. 5).

**Fig. 6:**
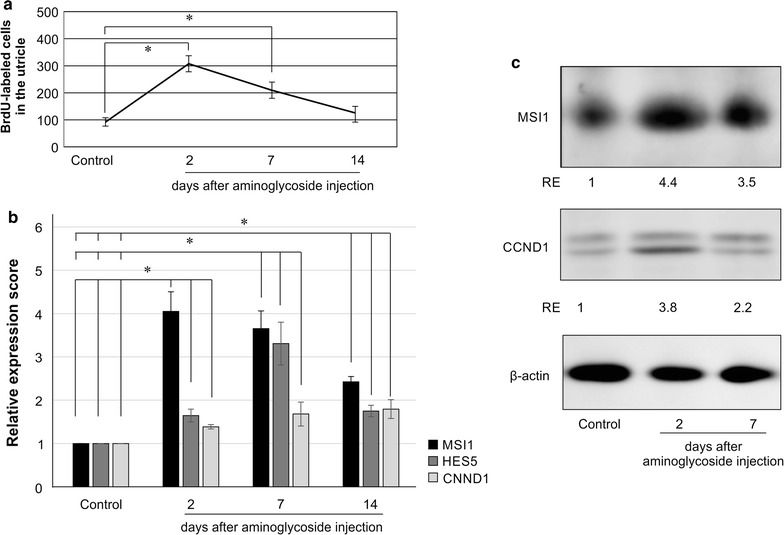
Ototoxic damage induced DNA synthesis and the expression of MSI1, HES5, and CNND1 in the vestibular sensory epithelium. Seven-day-old chickens received daily intramuscular injections of gentamicin sulfate (300 mg/kg) for 4 consecutive days, and utricles were then analyzed for BrdU incorporation and gene expression. **a** BrdU labeling in the sensory epithelium after aminoglycoside treatment. **b** Relative mRNA expression of MSI1, HES5, and CNND1 on days 2, 7, and 14 after the last aminoglycoside injection. mRNA levels in the treated chickens were normalized to those in untreated 12-day-old chickens as a control; *p < 0.05. **c** Western blotting analysis showing that MSI1 and CNND1 proteins were upregulated in the sensory epithelium during cell proliferation in the damaged inner ear. RE, relative expression normalized to the control sample

Aminoglycoside injection damaged the sensory epithelium of the utricles, as shown by microscopy analysis of hematoxylin–eosin–stained sections (Additional file 2: Fig. S1). In the treated utricles, the number of BrdU-labeled cells in the sensory epithelium was upregulated on days 2 and 7 after injection of aminoglycoside (Fig. 6a; Additional file 2: Fig. S2), indicating induction of cell proliferation after ototoxic stress.

Furthermore, the expression of MSI1, HES5, and CNND1 mRNAs was upregulated on days 2, 7, and 14 postinjection (Fig. 6b), corresponding to increased levels of the MSI1 and cyclin D1 proteins (Fig. 6c). These data suggested that regenerative processes, such as cell proliferation, occurred in parallel with the induction of MSI1 and associated genes in the inner ear of chickens after damage by toxic insults.

## Discussion

In neonate mammals, auditory cells retain the capacity for proliferation and regeneration; however, in mature mammals, sensory HCs cannot spontaneously regenerate, and the resulting hearing loss is permanent [5, 13]. In contrast, in birds and other lower vertebrates, the inner ear can completely regenerate lost HCs and recover the auditory function [14]. The restoration of the HC population in the avian ear can occur following the transformation of existing or proliferating SCs into HCs [5]; therefore, elucidation of the molecular mechanisms underlying HC regeneration through SC conversion would provide a platform for therapeutic strategies, including genetic manipulation, aimed at hearing loss restoration [15]. Accordingly, in this study, we performed gene expression profiling of SCs and HCs in the inner ear of chickens to identify differentially expressed genes and signaling pathways involved in spontaneous regenerative processes occurring in avian species. To the best of our knowledge, this is the first study comparing gene expression in SCs and HCs of the avian utricle.

Our microarray analysis showed that many genes described as HC markers [16] were upregulated in HCs, while no such genes were induced in SCs. At the same time, cell cycle-associated genes were highly expressed in normal SCs, indicating that potential HC progenitors were continuously proliferating in the chick utricle. These results are consistent with previous LCM-based studies on cell-specific markers in the inner ear of mice and humans [17].

In our investigation of the molecules playing an important role in the maintenance of HC progenitors in the inner ear, we focused on the MSI and HES genes. MSI1 is an RNA-binding protein and adult stem cell marker identified in several tissues, including the nervous system, colon, small intestine, mammary glands, and retina [11, 12]. MSI1 induces the Wnt pathway and mitogen-activated protein kinase (MAPK) signaling [10, 18] and functions as a translational repressor of Numb, a negative regulator of the Notch pathway, and p21Cip1, a cyclin-dependent kinase inhibitor, thus maintaining the proliferation of multipotent neural stem cells [19]. In postmitotic differentiated neurons, MSI1 expression is rapidly downregulated [20]. However, the information about MSI1 activity in the inner ear is limited [21 **].** MSI2 is known to work cooperatively with MSI1 during proliferation and maintenance of CNS stem cell populations [22]. Recently, it was also elucidated that MSI2 regulates hematopoietic stem cells and is linked with acute and chronic myeloid leukemia [23]. However, knowledge about the role of MSI2 in the inner ear is limited. Consistent with the role of MSI1 in supporting adult stem populations, our microarray data indicated that the expression levels of MSI1, MSI2, and downstream genes were increased in SCs, and these data were validated by quantitative RT-PCR. Moreover, the expression levels of Notch downstream transcription factors (HES5, HES1, HEY1, and HEY2) known to be regulated by MSI1 were elevated in SCs, further confirming the role of MSI1 and the Notch pathway in the maintenance of SCs in the chicken utricle. However, we could not detect Notch1 and recombining binding protein suppressor of hairless (RBPJ), which are key molecules involved in the Notch signaling pathway, or members of the Wnt pathway due to the poor quality of the corresponding data.

We also found that aminoglycoside treatment induced DNA synthesis indicative of cell proliferation in the utricle; this effect corresponded to the upregulation of MSI1 expression at the mRNA level in SCs and at the protein level in the sensory epithelium. Consistent with these results, the expression of HES5, which is implicated in the maintenance of neural stem and progenitor cells, and cyclin D1, a positive regulator of cell cycle progression, was also induced. Overall, these data suggested that MSI1 was involved in spontaneous cell regeneration in the chicken inner ear. In the avian basilar papilla, Atoh1, a transcription factor involved in the Notch signaling pathway, promotes the transdifferentiation of SC progenitors into HCs without mitosis in the early regeneration stage after damage [24]. In this study, ATOH1 expression in SCs was significantly elevated during regeneration after aminoglycoside injection (Additional file 2: Fig. S3), suggesting the induction of SC conversion into HCs. Other signaling molecules regulate the late mitotic stage of HC regeneration by stimulating cell cycle progression and proliferation of SCs. For example, the expression of p27kip1, a cyclin-dependent kinase inhibitor which blocks reentry into the cell cycle, was downregulated in a subgroup of the SCs that gave rise to HCs in gentamicin-damaged chicken cochleae [25], but upregulated in postmitotic mammalian SCs [5]. Moreover, adenovirus-mediated overexpression of S-phase kinase-associated protein 2 (Skp2), a repressor of p27kip1, stimulates DNA synthesis in the auditory epithelium [26]. These findings suggest that simultaneous delivery of genes such as ATOH1 and SKP2 may promote both stages of HC regeneration in the damaged sensory epithelium. However, the proliferation rate of HC precursors is likely to be insufficient for complete functional recovery. In our study, the induction of MSI1, a key regulator of the Notch pathway, which is important for the maintenance of stemness in the inner ear [5], corresponded to the upregulation of cell cycle inducers and DNA synthesis and thus may be a potential target of gene therapy approaches for managing inner ear defects. Further functional studies are needed for comprehensive analysis of MSI1 functional activity in the inner ear and validation of its potential in regenerative therapy for vestibular defects in humans.

Our microarray data also suggested that the proteoglycans decorin (DCN) and emphycan (EPYC), which were significantly upregulated in SCs compared with that in HCs, may also be targets for gene therapy approaches. Proteoglycans are the main components of connective tissue and are involved in multiple cell signaling pathways [27]. For example, DCN inhibits neural stem/progenitor cell differentiation into neurons [28] and supports axonal regeneration through interaction with cell adhesion molecules and growth factors in the central nervous system [29], although its role in the proliferation of tumor cells is controversial [30]. However, the modulatory activity of proteoglycans in cell proliferation, growth, and differentiation cannot be separated from their structural role in tissue organization, and comprehensive analysis is required to determine their regulatory functions in the maintenance of stemness and regeneration in the inner ear.

Among the genes involved in cell stemness and upregulated in SCs, we also identified SOX5 and SOX10, members of the Sox family of transcription factors. Sox proteins are critical players in embryogenesis and cell fate determination. Indeed, SOX10 promotes the survival of early cochlear progenitors during inner ear development. The function of SOX5 in the inner ear remains unknown. However, SOX5, which is part of the SOXD transcription factor subfamily, is critically involved in multiple aspects of neuronal differentiation for subsets of cortical neurons and has been shown to regulate chondrogenesis [31, 32]. A recent study showed that the expression of another Sox protein, Sox2, is induced in supporting cells proliferating in embryonic chicken cochleae in response to gentamicin treatment [25]. These findings strongly suggest a role of Sox-mediated transcription in supporting auditory function, which should be examined in detail in future studies.

## Conclusions

In conclusion, this is the first study to perform gene expression profiling of SCs in the avian utricle using LCM and cDNA microarray. Our data underscore the importance of the Notch pathway in the biological functions of SCs and regenerative events in the inner ear. Our findings suggested that MSI1 and its downstream signaling molecules may play an important role in HC regeneration in the inner ear in both normal and damaged states. Most importantly, we showed, for the first time, that the adult stem cell marker MSI1 may play a significant role in regenerative processes in the inner ear both during aging and after ototoxic damage, suggesting that MSI1 may be a potential candidate target of future therapeutic approaches to treat vestibular dysfunction.

## Additional files

**Additional file 1:** **Table S1.** Primers used for quantitative RT-PCR. **Table S2.** Genes upregulated more than fivefold in hair cell s than in supporting cells. **Table S3.** Genes upregulated more than 2.5-fold in supporting cells than in hair cells.

**Additional file 2:** **Fig. S1.** Representative images of hematoxylin–eosin–stained sections of the sensory epithelium from chicken utricles. (A) Epithelium from a control (saline-injected) chicken. (B) Epithelium from a gentamicin sulfate-injected chicken on day 14 postinjection. Extruded hair cells (arrowheads) and phagocytosis (arrows) are indicated. **Fig. S2.** Representative images of BrdU incorporation in the utricle as analyzed by immunohistochemistry in control chickens (A) and aminoglycoside-treated chickens on days 2 (B), 7 (C), and 14 (D) after the last gentamicin injection. **Fig. S3** Relative expression of the ATOH1 gene. Increased ATOH1 mRNA levels were detected in supporting cells 3 h after aminoglycoside injection and continued for the next 14 days (*p < 0.05).

## Abbreviations

EDTA: ethylenediaminetetraacetic acid
HCs: hair cells
LCM: laser capture microdissection
MBP: myelin basic protein
MSI: musashi
PBS: phosphate-buffered saline
RT-PCR: reverse transcription polymerase chain reaction; SCs: supporting cells
