# Supplementary material for: Musashi-1 is the candidate of the regulator of hair cell progenitors during inner ear regeneration
Source: BMC Neurosci. 2017 Aug 16;18:64. doi: 10.1186/s12868-017-0382-z (PMC5559865; doi:10.1186/s12868-017-0382-z)
Supplement: Supplementary file 1 — Additional file 1: Table S1. Primers used for quantitative RT-PCR. Table S2. Genes upregulated more than fivefold in hair cell s than in supporting cells. Table S3. Genes upregulated more than 2.5-fold in supporting cells than in hair cells. [file 12868_2017_382_MOESM1_ESM.pdf]

Supplementary Tables

Musashi-1 regulates the proliferation of hair cell progenitors during regeneration in the inner ear

Takahiro Wakasaki, Hiroaki Niiro, Siamak Jabbarzadeh-Tabrizi, Mitsuru Ohashi, Takashi Kimitsuki, Takashi Nakagawa, Shizuo Komune, Koichi Akashi

Table S1. Primers used for quantitative RT-PCR.

Gene	Forward primer	Reverse primer	Probe	Product size (bp)
**Taqman**				
HES5	AAGCTGGAGAAAGCCGACATC	TTCTTTGAGGCACCAGGCATA	FAM-TACAGCCGAGCTTTTGCAGCCTCTG-TAMURA<br>FAM-ACGAGACGCTGCAGATGGCGC-TAMURA	126
Atoh1	AACAACGACAAGAAGCTCTCCAA	GCGAGGGCGCTGATGTAG	FAM-CGCCCGTCGCTACTACCGATTGG-TAMURA	68
18S	AGTCCCTGCCCTTTGTACACA	GATCCGAGGGCCTCACTAAAC		69
**SYBR Green**				
Musashi-1	GCTTCCAAGCTGCCACCTAC	TCCGCTCTACACGGAACTCA		90
Musashi-2	TTGGTTGCCATCTCACTTGG	GGTCGTTTGCTGTTGGTGAA		129
RBPJ	GATGTGGAAGCCGAAACCAT	CTCCAACCCTCTCGAAATGC		86
HES1	GGTGAAGCACCTGAGGAACC	TTGAAACCAGCCCGGTATTT		93
CCND1	GTGGTAGCAGCTGTGCAAGG	TCTTGGCAGGCTCGTAAACA		125
DLK1	GTGCCCAGGGATTTACAGGA	CTCCATTCTGGCAGGGAGAG		81
MYO6	AGTGTTTCCTGCCGAAGAGG	TGTGGAGGAGAGTGGCTTCA		86
EPYC	AACTGCCTCACCCATTTGTC	CTGTCAACGGTGCAGAAGAA		117
DCN	AGTGTCATCTTCGCGTTGTG	TCCAGCAGAGTTGTGTCAGG		85
GAPDH	GACGTGCAGCAGGAACACTA	CTTGGACTTTGCCAGAGAGG		112

Table S2. Genes upregulated more than 5-fold in hair cell s than in supporting cells.

Gene	Fold change	RefSeq transcript ID	Gene	Fold change	RefSeq transcript ID
fibrinogen beta chain	37.31	XM_420369	solute carrier family 9	6.85	NM_001031305
delta/notch-like EGF repeat containing	19.65	XM_422604	Fas apoptotic inhibitory molecule 2	6.80	XM_424507
calsyntenin 1	16.29	XM_417603	adenylate cyclase activating polypeptide 1	6.76	NM_001001291
BR serine/threonine kinase 2	13.51	XM_421031	matrilin 4	6.67	XM_425698
nuclear receptor subfamily 1, group H, member 4	13.50	NM_204113	neuronal pentraxin II	6.58	XM_414750
fibronectin type III domain containing 7	12.77	XM_422182	EPH receptor A5	6.49	NM_205105
IQ motif containing with AAA domain	11.03	XM_421878	leucine rich repeat containing 17	6.49	XM_415965
diacylglycerol kinase, iota	10.44	XM_416357	myosin binding protein C, slow type	6.29	XM_416332
mannose-binding lectin (protein C) 2, soluble	9.01	NM_204349	calcium binding protein 39-like	6.25	NM_001006272
neuroplastin	9.01	XM_413703	calcium binding protein 39-like	6.25	NM_001006272
chromogranin A (parathyroid secretory protein 1)	8.77	XM_421330	synaptosomal-associated protein, 25kDa	6.25	NM_205458
hydrocephalus inducing homolog (mouse)	8.77	XM_425112	otoferlin	6.10	XM_420015
secretogranin III	8.55	XM_413807	calcium binding protein 39-like	5.95	NM_001006272
			synaptosomal-associated protein, 25kDa	5.92	NM_205458
NFASC, transcript variant 2, mRNA.; isoform 3	8.26	NM_001004394; NM_001004493; NM_001004709	potassium voltage-gated channel, shaker-related subfamily, beta member 1	5.88	NM_204906
			ATPase, Ca++ transporting, plasma membrane 2	5.78	XM_414301
potassium voltage-gated channel, shaker-related subfamily, member 10	8.00	NM_204462	bone morphogenetic protein 2	5.78	NM_204358
diacylglycerol kinase, iota	7.63	XM_416357	insulin-like growth factor 2 receptor	5.78	NM_204970
titin	7.63	XM_421979	coagulation factor XIII, A1 polypeptide	5.75	NM_204685
nuclear factor related to kappaB binding protein	7.52	NM_001006317	cadherin 2, type 1, N-cadherin (neuronal)	5.71	NM_001001615
hexosaminidase A (alpha polypeptide)	7.41	NM_001030390	doublecortin-like kinase 2	5.71	XM_420439
titin	7.41	XM_421979	R-spondin 3 homolog (Xenopus laevis)	5.65	XM_419752
brain-derived neurotrophic factor	7.30	NM_001031616	N-acetylglucosaminyltransferase VI	5.62	NM_204681

lipoxygenase homology domains 1	7.25	XM_425221	proprotein convertase subtilisin/kexin type 2	5.29	XM_419332
adenylate cyclase activating polypeptide 1 (pituitary)	7.04	NM_001001291	delta/notch-like EGF repeat containing	5.21	XM_422604

Table S3. Genes upregulated more than 2.5-fold in supporting cells than in hair cells.

Gene	Fold change	RefSeq transcript ID	Gene	Fold change	RefSeq transcript ID
fibrinogen beta chain	37.31	XM_420369	solute carrier family 9 (sodium/hydrogen exchanger), member 9	6.85	NM_001031305
delta/notch-like EGF repeat containing	19.65	XM_422604	Fas apoptotic inhibitory molecule 2	6.80	XM_424507
calsyntenin 1	16.29	XM_417603	adenylate cyclase activating polypeptide 1 (pituitary)	6.76	NM_001001291
BR serine/threonine kinase 2	13.51	XM_421031	matrilin 4	6.67	XM_425698
nuclear receptor subfamily 1, group H, member 4	13.50	NM_204113	neuronal pentraxin II	6.58	XM_414750
fibronectin type III domain containing 7	12.77	XM_422182	EPH receptor A5	6.49	NM_205105
IQ motif containing with AAA domain	11.03	XM_421878	leucine rich repeat containing 17	6.49	XM_415965
diacylglycerol kinase, iota	10.44	XM_416357	myosin binding protein C, slow type	6.29	XM_416332
mannose-binding lectin (protein C) 2, soluble	9.01	NM_204349	calcium binding protein 39-like	6.25	NM_001006272
neuroplastin	9.01	XM_413703	calcium binding protein 39-like	6.25	NM_001006272
chromogranin A (parathyroid secretory protein 1)	8.77	XM_421330	synaptosomal-associated protein, 25kDa	6.25	NM_205458
hydrocephalus inducing homolog (mouse)	8.77	XM_425112	otoferlin	6.10	XM_420015
secretogranin III	8.55	XM_413807	calcium binding protein 39-like	5.95	NM_001006272
neurofascin; isoform 2 precursor is encoded by transcript variant 2; Gallus gallus neurofascin (NFASC), transcript variant 2, mRNA.; isoform 3 precursor is encoded by transcript variant 3; Gallus gallus neurofascin (NFASC), transcript variant 3, mRNA.	8.26	NM_001004394; NM_001004493; NM_001004709	synaptosomal-associated protein, 25kDa	5.92	NM_205458
			potassium voltage-gated channel, shaker-related subfamily, beta member 1	5.88	NM_204906
			ATPase, Ca++ transporting, plasma membrane 2	5.78	XM_414301
potassium voltage-gated channel,	8.00	NM_204462	bone morphogenetic protein 2	5.78	NM_204358

shaker-related subfamily, member 10					
diacylglycerol kinase, iota	7.63	XM_416357	insulin-like growth factor 2 receptor	5.78	NM_204970
titin	7.63	XM_421979	coagulation factor XIII, A1 polypeptide	5.75	NM_204685
nuclear factor related to kappaB binding protein	7.52	NM_001006317	cadherin 2, type 1, N-cadherin (neuronal)	5.71	NM_001001615
hexosaminidase A (alpha polypeptide)	7.41	NM_001030390	doublecortin-like kinase 2	5.71	XM_420439
titin	7.41	XM_421979	R-spondin 3 homolog (Xenopus laevis)	5.65	XM_419752
brain-derived neurotrophic factor	7.30	NM_001031616	N-acetylglucosaminyltransferase VI	5.62	NM_204681
lipoxygenase homology domains 1	7.25	XM_425221	proprotein convertase subtilisin/kexin type 2	5.29	XM_419332
adenylate cyclase activating polypeptide 1 (pituitary)	7.04	NM_001001291	delta/notch-like EGF repeat containing	5.21	XM_422604
Fas apoptotic inhibitory molecule 2	6.99	XM_424507	heat shock protein 90kDa beta (Grp94), member 1	5.13	NM_204289
glypican 4	6.90	XM_420222	adhesion molecule with Ig-like domain 2	5.10	XM_416052
serpin peptidase inhibitor, clade I (neuroserpin), member 1	6.85	NM_001004411	slit homolog 3 (Drosophila)	5.03	XM_414503
