# Supplementary material for: Musashi-1 is the candidate of the regulator of hair cell progenitors during inner ear regeneration
Source: BMC Neurosci. 2017 Aug 16;18:64. doi: 10.1186/s12868-017-0382-z (PMC5559865; doi:10.1186/s12868-017-0382-z)
Supplement: Supplementary file 2 — Additional file 2: Fig. S1. Representative images of hematoxylin–eosin–stained sections of the sensory epithelium from chicken utricles. (A) Epithelium from a control (saline-injected) chicken. (B) Epithelium from a gentamicin sulfate-injected chicken on day 14 postinjection. Extruded hair cells (arrowheads) and phagocytosis (arrows) are indicated. Fig. S2. Representative images of BrdU incorporation in the utricle as analyzed by immunohistochemistry in control chickens (A) and aminoglycoside-treated chickens on days 2 (B), 7 (C), and 14 (D) after the last gentamicin injection. Fig. S3 Relative expression of the ATOH1 gene. Increased ATOH1 mRNA levels were detected in supporting cells 3 h after aminoglycoside injection and continued for the next 14 days (*p < 0.05). [file 12868_2017_382_MOESM2_ESM.pdf]

Supplementary Figures

Musashi-1 is the candidate of the regulator of hair cell progenitors during inner ear regeneration

Takahiro Wakasaki, Hiroaki Niiro, Siamak Jabbarzadeh-Tabrizi, Mitsuru Ohashi, Takashi Kimitsuki, Takashi Nakagawa, Shizuo Komune, Koichi Akashi

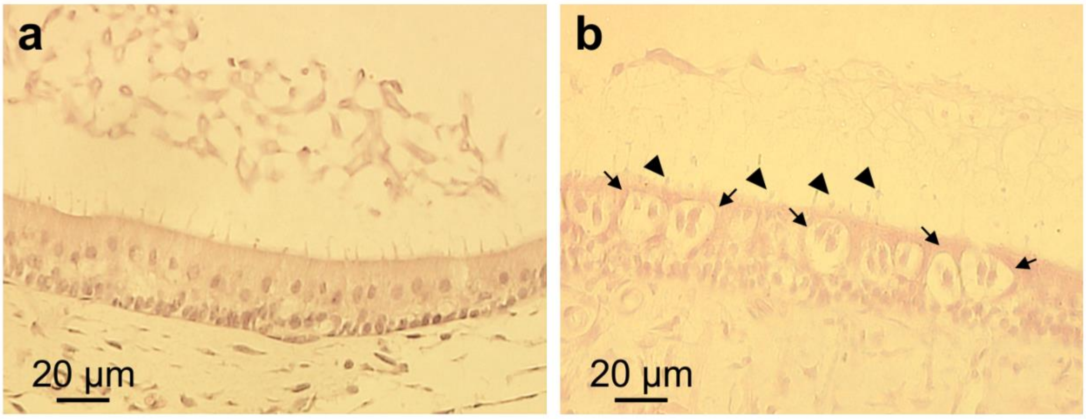

Fig. S1. Representative images of hematoxylin-eosin-stained sections of the sensory epithelium from chicken utricles. (A) Epithelium from a control (saline-injected) chicken. (B) Epithelium from a gentamicin sulfate-injected chicken on day 14 postinjection. Extruded hair cells (arrowheads) and phagocytosis (arrows) are indicated.

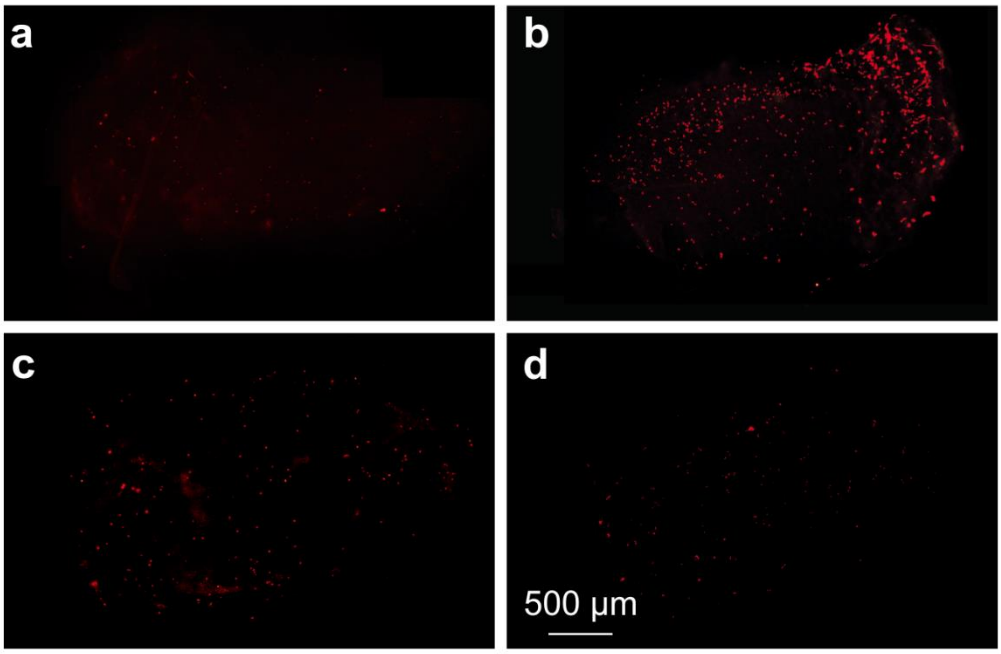

Fig. S2. Representative images of BrdU incorporation in the utricle as analyzed by immunohistochemistry in control chickens (A) and aminoglycoside-treated chickens on days 2 (B), 7 (C), and 14 (D) after the last gentamicin injection.

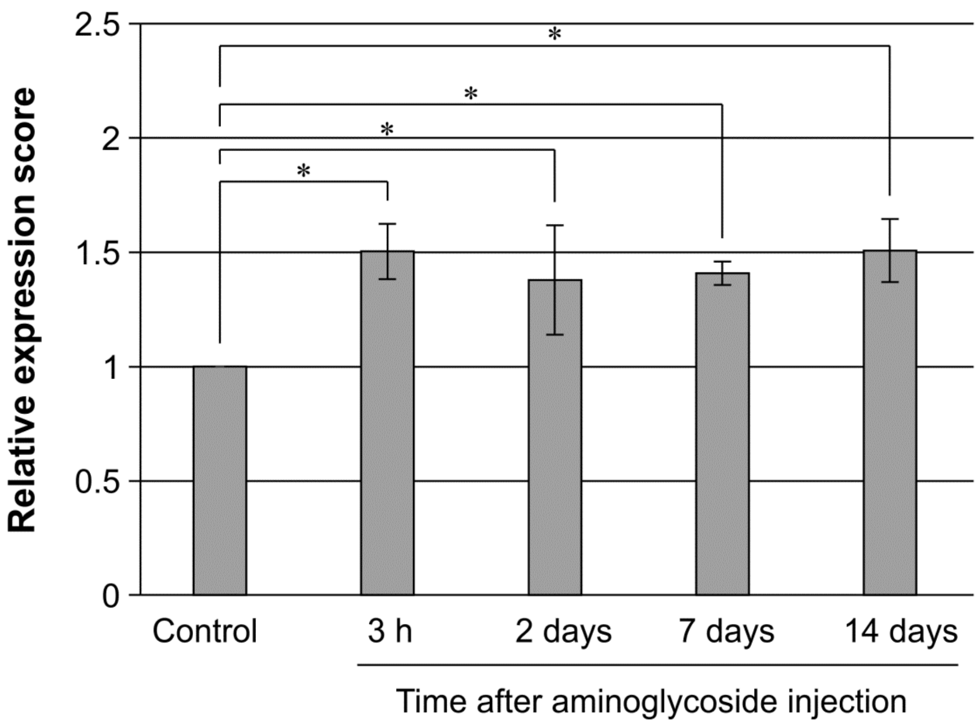

Fig. S3. Relative expression of the ATOH1 gene. Increased ATOH1 mRNA levels were detected in supporting cells 3 h after aminoglycoside injection and continued for the next 14 days (*$p < 0.05$).
